# Contracting Out Non-State Providers to Provide Primary Healthcare Services in Tanzania: Perceptions of Stakeholders

**DOI:** 10.15171/ijhpm.2018.46

**Published:** 2018-05-22

**Authors:** Stephen Maluka

**Affiliations:** Institute of Development Studies, University of Dar es Salaam, Dar es Salaam, Tanzania.

**Keywords:** Contracting Out, Non-State Providers, Primary Healthcare, Tanzania

## Abstract

**Background:** In the attempt to move towards universal health coverage (UHC), many low- and middle-income countries (LMICs) are actively seeking to contract-out non-state providers (NSPs) to deliver health services to a specified population. Research on contracting-out has focused more on the impact of contracting-out than on the actual processes underlying the intervention and contextual factors that influence its performance. This paper reports on perceptions of stakeholders on contracting-out faith-based hospitals through service agreements (SAs) to provide primary healthcare services in Tanzania.

**Methods:** We adopted a qualitative descriptive case study design. Qualitative research tools included document review and in-depth interviews with key informants, and data were analysed using a thematic approach.

**Results:** Stakeholders reported mixed perceptions on the SA. The government considered the SA as an important mechanism for improving access to primary healthcare services where there were no public hospitals. The faith-based hospitals viewed the SA as a means of overcoming serious budget and human resource constraints as a result of the tightening funding environment. However, constant delays in disbursement of funds, mistrust among partners, and ineffective contract enforcement mechanisms resulted into negative perceptions of the SA.

**Conclusion:** SAs between local governments and faith-based hospitals were perceived to be important by both parties. However, in order to implement SAs effectively, the districts should diversify the sources of financing the contracts. In addition, the government and the faith-based organizations should continually engage in dialogue so as to build more trust between the partners involved in the SA. Furthermore, the central government needs to play a greater role in building the capacity of district and regional level actors in monitoring the implementation of the SA.

## Background

Global attention has recently converged on the need for countries to achieve universal health coverage (UHC), which aims to guarantee access to healthcare services without facing financial ruin.^1^ In the attempt to move towards UHC, many low- and middle-income countries (LMICs) are actively seeking to increase the contributions of the non-state providers (NSPs). NSPs include all providers who exist outside the public sector.^2^ Some of the key NSPs in LMICs are faith-based organisations (FBOs). One of the most common approaches to engage NSPs has been to contract-out the delivery of health services to a specified population on behalf of the government.^3^ A contract document usually specifies the responsibilities of the parties to the contract, the precise range of services to be provided, time span, the performance standards to be achieved, procedures for performance monitoring, and terms of payment and costs.^4^

Advocates of contracting-out argue that given the resource-constraints of existing health systems, a more realistic approach to improving access to healthcare is to acknowledge and build upon the opportunities and resources of the NSPs.^4-7^ However, critics claim that, in the context of many developing countries, contracting-out may be unlikely to achieve its intended objectives for several reasons. These include high administrative costs, lack of sufficient providers for meaningful competition in many rural areas and the power of vested interests, which may try to gain control over the contracting process.^4,8^ Others are concerned about user fees associated with private health services. They maintain that increasing the role of the private sector limits the use of healthcare among the poorest, who cannot afford the services, consequently reducing access and equity in the use of healthcare.^8-10^

In Tanzania, the private not for profit sector – of which the faith-based facilities make up the overwhelming majority – is the second largest provider of healthcare in the country after the public sector.^11^ Although in Tanzania NSPs existed since the colonial time, the contract between the government and the NSPs was mainly founded on relational contracts.^12^ The Government formally negotiated the hospital agreements in 1992 with faith-based facilities.^12^ The decentralisation policy led to the 2005 revision of the 1992 Agreements so that the contracts may be signed at the district level. Subsequently, in 2007 a new type of operational contract known as the service agreement (SA) was introduced. The SA is a contractual agreement between the government and health service providers that defines duties and responsibilities of both parties. The SA requires districts to enter into formal contract not just to increase services, but also to bring services close to the people and at an affordable cost. The districts have authority to decide which type of the health facility to be contracted based on the needs of the districts. The health facilities could be hospitals, health centres or dispensaries. Since its endorsement and the signing of the first SA in 2008, more District Councils have entered into agreements with health facilities owned by non-state healthcare providers. By 2012, already 37 districts had entered into SAs with NSPs.^13^

Several reviews of the literature have been already carried out on contracting-out experiences in developing countries. The first review which reported experiences of contracting out non-clinical services in Southern Africa, found mixed evidence of benefits of contracting-out to private providers.^14^ The authors reported that in some countries such as Zimbabwe and South Africa, contracted providers could provide services of the same or higher quality at lower cost, while in Ghana and Tanzania, there were no significant differences in the performance between contracted and public providers.^14^ A second review focused on the capacity of contracting out strategies to benefit the poorest, and underlined the lack of robust evidence in that respect.^15^ A third review concluded that contracting out could be very effective and should be expanded, with more rigorous evaluations.^16^ Further to that, other reviews suggest that while contracting out has improved access to health services, the effects on other performance dimensions such as quality of services, efficiency and equity remain unknown.^17,18^

In Tanzania, earlier assessments of the previous contracts have been done. These assessments reported that misperceptions and unfamiliarity hampered utilization of possible synergies.^11,12,19,20^ However, these studies focused on a different form of contracts which were negotiated and signed centrally by the Ministry of Health (MoH). This paper specifically addresses the new district contracts. The paper elucidates perceptions on contracting-out faith-based hospitals for the provision of primary healthcare services in Tanzania. Understanding the perceptions of stakeholders is crucial for proper implementation of the SA between the government and NSPs and could, in turn, help to assess the feasibility and sustainability of the SA.

## Methods

### Study Design and Settings

This study adopted a case study approach.^21^ The case study approach permitted the rigorous analysis of the SA between the districts and NSPs within its real-life context. The approach also made it possible to explore multiple perspectives of various stakeholders on the SA. Four district councils were purposively selected for in-depth analysis. The districts were selected from different health zones in Tanzania: Lushoto (Northern), Kilwa (Southern), Singida rural (Central), and Iringa (Southern highlands). Tanzania operates a decentralised health system, organized around three functional levels: districts (primary level), regional (secondary level), and referral hospitals (tertiary level). Within the framework of the decentralisation process, regions and districts have full responsibilities for delivering health services within their areas of jurisdiction, and report administratively to the President’s Office Regional Administration and Local Government (PO-RALG).

Dispensary is the lowest level of healthcare delivery in the country, and is ideally run by a clinical assistant aided by an enrolled nurse; and offers basic outpatient curative care to between 6000 and 10 000 people. Health centres serve populations of about 50 000. Health centres are normally run by the Clinical officers supported by enrolled nurses. Most districts in Tanzania have a government-run district hospital; others rely on faith-based hospitals and they become designated district hospitals (DDHs) which are eligible to receive government subsidies. Several districts are grouped into a region, each of which has a regional hospital. This study focused on the SAs created and signed between the district authority and the faith-based hospitals. Table 1 provides a summary of the key demographic and health characteristics in the four study districts.

**Table 1:** Key Demographic and Health Characteristics of the Study Districts

**Key Indicator**	**Iringa**	**Ikungi**	**Lushoto**	**Kilwa**
Population	254 032	272 959	332 436	200 015
Population growth rate (%)	1.6	2.4	1.1	0.9
Hospitals	1	2	2	2
Health centres	6	3	5	5
Dispensaries	61	32	46	47

Source: District annual health plans (2017/2018); Census Report 2012.

### Data Collection Techniques

To explore the perceptions of various stakeholders on the SA, we conducted in-depth interviews with key informants, including Council Health Services Board (CHSB), District Medical Officers (DMOs), the Council Officials, health facility owners and management teams of the health facilities. Interviews were also conducted with national and regional level stakeholders. As indicated in Tables 2 and 3, a total of 38 interviews were carried out. Purposive and snow ball sampling techniques were used to recruit respondents. Interviews were carried out until saturation point was reached, meaning that no new concepts were identified in successive interviews. Oral consent was obtained from all those who participated in the interviews. All interviews were audio-recorded after obtaining verbal permission from the respondents.

**Table 2:** District Level Respondents

**District Level Respondents**	**No. of the Interviews**			
	**Singida**	**Lushoto**	**Iringa**	**Kilwa**
CHMT	3	3	4	3
The CHSB	1	-	1	1
Diocese Leaders (Bishops’ offices)	1	2	1	1
District legal officers	1	-	-	1
Health facility providers and hospital administrators	2	2	2	2
Hospital financial officers	**-**	1	1	-
Total key informants	8	8	9	8

Abbreviations: CHSB, Council Health Services Board; CHMT, Council Health Management Team.

**Table 3:** National and Reginal Level Respondents

**Category of Respondent**	**Role in the SA**	**Number**
MoH, Community Development Gender, Elderly and Children (former Ministry of Health & Social Welfare)	• Formulates the SA template (policy) and monitors the implementation of this policy • Finances SAs	1
Umbrella Organizations - Involve the association of institutions with common interest in issues related to health	• They offer technical support to health facilities under their umbrella that have entered into SAs with the district councils	1
Development partners	• Provide technical and financial support in the development and implementation of SAs	1
RHMT	• Provide technical back up to the district councils in the implementation of SAs	2
**Total Key Informants**		5

Abbreviations: SA, service agreement; RHMT, Regional Health Management Team; MoH, Ministry of Health.

In addition, various documents including the SA template, the signed SAs, and hospital annual reports were reviewed. The documents were meant to supplement and cross-check information on the nature and content of the SA including the type of services the contract covers, the target population, the specification of performance requirements, and contract payment mechanisms.

### Data Analysis

Individual interviews were analysed for themes in three steps.^22^ First, the notes from key informant interviews were manually open coded. Next, the codes and coding trees were reviewed and refined by going back and forth between the codes and the transcripts. In the third step, the codes of all interviews were organized into themes. The categorization of themes was extensively discussed among research team members in group meetings until consensus was achieved. Finally, data were summarised and synthesised, retaining as much as possible the key expressions of respondents. Responses from different types of respondents were compared to see where there were differences and similarities.

## Results

This section presents the findings from the analysis of key informant interviews with key stakeholders involved in the formulation and implementation of the SA between the district government and faith-based hospitals. Documentary data were used to support, verify and highlight the key issues that emerged. Verbatim quotes from the respondents have been included to illustrate the main messages communicated.

### The Service Agreement Mechanism

This section describes the SA mechanism in Tanzania. It describes factors that led to the creation of the contract, the process of creating and signing the contract, types of services covered, payment mechanisms and monitoring of the implementation of the contract.

#### Why Did Districts Create Service Agreements?

The demand for the SA emerged from both the government and the FBOs. On the side of the government, there was desire to increase access to healthcare services to the population particularly in rural and hard to reach areas where the public health facilities were not available. The central government had established a policy of providing free maternal and child health services as part of achieving the Millennium Development Goals 4 and 5. Districts were, therefore, required to provide free maternal and child health services. The lack of public hospitals forced the districts to negotiate with the faith-based hospitals to provide free maternal and child health services and subsidise user fees for the remaining services. The contracted faith-based hospitals became DDHs.

“*We needed to have a district hospital, and because our colleagues already had a hospital which had the right qualities of being a district hospital, we saw that it would be better if we started the process of entering into the SA*” (Interview with a district health manager).

On the side of the faith-based hospitals, there were increased demands for health professionals and declining financial support from the donors. It was evident from the analysis of the interviews with both district health managers and FBOs that during the early 2000s, most of the faith-based hospitals faced challenges in providing health services due to shortage of financial and human resources. Donors had significantly reduced financial support to the faith-based hospitals. In addition, the government had improved salaries and incentives for health personnel working in the public health facilities. As a result, medical doctors and nurses were moving away from the faith-based hospitals to the public hospitals. The faith-based hospitals could not generate enough resources for medicine and medical supplies, equipment, renovation of infrastructures as well as paying salaries to the health providers. Given this situation, the faith-based hospitals needed assistance from the government.

*“At that time, we longed to become a District Designated Hospital (DDH) after seeing that the hospital had expanded and that there would be problems in paying our staff according to Government scales. We were used to paying our staff according to what we used to get. Considering the fact that we had qualified to become a DDH, we decided to apply for the status so as to provide services even better*” (Interview with a diocese leader).

This was confirmed by the district and regional health managers as elaborated by one of them below:

“*But even our colleagues on the other side had budget and human resource constraints. The donors had already withdrawn, and the staff were quitting the job from the religious hospitals to Government hospitals. As such they also started losing staff, besides the fact that they had many buildings and enough equipment. So they too realized that if they entered into this SA, they would revitalize”* (Interview with a district health manager).

Furthermore, there was pressure from international actors to engage NSPs in order to accelerate efforts towards UHC. The analysis of documents and interviews revealed that a number of international actors played very crucial role in advocating the creation and implementation of SAs. The key international actors were: the Tanzania Germany Programme for Health Support (TGPHS) supported by the German Development Cooperation (GIZ and KfW), USAID and DANIDA. However, TGPHS seemed to play a leading role in the creation and implementation of SAs in Tanzania. For example, in two study districts, (Lushoto and Kilwa) the TGPHS played very critical role. Whereas KfW provided financial support to the districts to get into SAs, GIZ provided technical support through Tanzanian and international experts advising policy makers and implementers of health services alike. The GIZ also played very crucial role in building capacities of the involved parties to be able to effectively engage in discussions and exchange of experiences.

#### How Were Service Agreements Created and Signed?

The analysis of interviews and the signed SAs revealed that the districts had the mandate of creating and signing the contracts with NSPs, including private for profit. The key government actors at the district level were the Council Health Management Teams (CHMTs) and the District Executive Officer’s Office. On the part of the faith-based hospitals, the key actors involved were diocese leaders (owners of the faith-based hospitals) and the hospital administrators. The MoH was primarily responsible for the formulation of the generic SA template that was to be adapted by the districts. The Ministry also advocated the creation and signing of the contracts. The regional level provided technical back up to the district councils in the creation and signing of the contracts.

The district governments formed teams of experts to facilitate the process of creating and signing of SAs. The negotiation process was accompanied by a number of activities. The faith-based hospitals received technical support from the Christian Social Services Commission (CSSC), an umbrella organization for Christian faith based organisations in Tanzania. The districts mainly got technical support from the MoH and the international organisations mainly the Tanzania Germany Programme for Health Support.

The contracts were signed following successful negotiation between the two parties. The signatories from the local government side were chairperson of Council, District Executive Director in the presence of DMO and the legal officer of the council. From the faith-based contracted hospitals, the signatories were the diocese leaders and the hospital administrators.

#### Which Services Were Covered?

In all the four faith-based hospitals, the SA mainly aimed at providing free maternal and child health services. Specific services covered included: antenatal care, delivery and postnatal care services; and Prevention of Mother to Child Transmission of HIV (PMTCT). In addition, the contracted faith-based hospitals subsidised user fees to the general population residing in the district. However, in all the four districts, the contracted hospitals provided subsidised healthcare services to many other individuals from neighbouring districts which were not formally included in the contract.

#### How Were Service Agreements Financed?

In two districts, the initial SA was funded by the GIZ. The contracted faith-based hospitals were paid fee for services. The GIZ disbursed funds to the district authorities. The district authority paid the faith-based hospitals for services rendered to pregnant women, children and other vulnerable groups of the population. The contracted faith-based hospitals had to fill claim forms to be submitted to the district council for reimbursement in line with the agreed rates and conditions. However, after the GIZ had withdrawn financial support, the districts were not able to finance the contract using locally generated resources. Subsequently, the districts changed the mode of payment from fee for services to lump sum. The similar financing mechanism was reported in the other two districts which were not supported by the donors. The budget for the faith-based hospitals had to be included in the district annual health plans and financed by the central government using a basket fund. A basket fund is largely depended on the donor support through the central government. The contracted faith-based hospitals were required to receive 25%-30% of the district annual health plan budget of the respective district. The formula for allocating funds to the districts and the contracted faith-based hospitals was determined by the central government. This means that funds disbursed by the central government to the contracted faith-based hospitals varied across the districts. Funds were to be channelled from the central government to the contracted faith-based hospitals through the district councils on a quarterly basis. Analysis of documents revealed that if the costs of providing free maternal and child health services were more than the services provided by the contracted faith-based hospitals, it was the responsibility of the District Councils to top up any extra amount stipulated in the SA for maternal and child health services. However, interviews with all types of respondents revealed that the districts only depended on the central government funding to finance SAs.

#### What Were the Monitoring and Evaluation Mechanisms?

The central government had the responsibility of monitoring the progress of SAs implementation through quarterly technical, financial and progress reports. The faith-based hospitals were only eligible to receive funds following submission of quarterly technical, financial and progress reports. The central government also had mandate to conduct supervision visits to the districts occasionally. The Regional Health Management Team (RHMT) and the CHMT were responsible to oversee the technical implementation of SAs on behalf of the central government. The CHMT members had power to conduct spot checks in the contracted health facilities.

### Perceived Benefits of Service Agreements

Respondents reported mixed perceptions on SAs. The government considered the SA as an important mechanism for improving access to primary healthcare services where there were no public hospitals. It was evident that the contracted faith-based hospitals did not charge maternal and child health services. In addition, the costs of other services were relatively lower than in other faith-based hospitals.

“*By having this hospital, many people are able to get treatment right here. Maternal and child health services are not charged. Even other patients are now treated at a reduced cost”* (Interview with a district health manager).

The faith-based hospitals viewed the SA as a means of overcoming serious budget and human resource constraints as a result of the tightening funding environment. The faith-based hospitals highly appreciated financial, human resource and other support provided by the central government as part of the SA.

*“The government is shouldering several costs, especially paying salaries and subsidies for medicines through the Medical Stores Department. To a large extent, it has reduced the challenge of paying salaries to staff”* (Interview with an accountant of a faith-based hospital).

Another respondent added:

*“Leaving aside the general financial benefit, we also benefit in other ways; some of our staff are paid salaries by the government. We also get staff who are seconded from the district, especially doctors and nurses”* (Interview with in-charge of a faith-based hospital).

On the part of the government, the SA made it possible for the districts to have hospital infrastructures owned by the FBOs instead of building new hospitals. All district health managers reported that before signing of the SA with the faith-based hospitals, referral cases from the health centres in their districts were sent to the regional hospitals which were far from the population they serve. After signing the SA, services have been brought closer to the people.

“*The presence of the agreement has helped to bring health services closer to the people. In the past, patients had to be referred to the regional hospital, even those coming near the hospital owned by the non-state care providers. After signing the agreement, patients go to this hospital for services*” (Interview with district health manager).

Another respondent added:

*“Firstly, we were legally obliged to have a district hospital. So by entering into this agreement, we got a district hospital. Secondly, we saw that our patients were facing a lot of difficulties going all the way to the regional hospital”* (Interview with district health manager).

Almost all government officials reported that following the implementation of the SA, there was increased utilization of maternal and child health services. The contracted faith-based hospitals confirmed that they had been receiving more clients for maternal and child health services following the implementation of the SA. Some of the clients were coming from neighbouring districts. Such a situation increased the workload of health staff as well as extra cost for these health facilities.

“*Since the signing of the SA, we have been receiving many patients. Some patients come from neighbouring districts which are not part of the SA. We are working at a loss because we are spending more than what we receive from the district*” (Interview with a faith-based hospital manager).

Another respondent added:

*“Running the hospital is becoming difficult because the number of patients has increased, and not all of them come from within our district. They come from other districts and regions*” (Interview with in-charge of a faith-based hospital).

### Perceived Barriers to the Implementation of Service Agreements

While almost all respondents acknowledged the importance of SAs between the districts and the faith-based hospitals, they raised several concerns as outlined hereunder.

#### Inadequate and Constant Delays in Financial Support

The Government was to disburse funds to the contracted hospitals on quarterly basis. However, it was noted that there were significant delays in the disbursement of funds to the hospitals. This affected delivery of quality healthcare services in the contracted faith-based hospitals.

“*The main challenge is delay in funds. The district cannot send funds to the hospital, if we have not received from the central government. At least now the flow of basket funds is good. There was time when funds could be delayed even for six months. The hospital had to borrow drugs from vendors or use other sources*” (Interview with district official).

Another respondent added:

*“The money from the government usually comes late. It may be the case that it is a problem from the top. But when it comes, we already have used our resources, and we get stuck in one way or another*” (Interview with in-charge of a faith-based hospital).

However, while the contracted faith-based hospitals felt that the funds provided by the government were inadequate, the district and regional health managers had a different view. According to our respondents, the FBOs had high expectations from the government. The government officials had the opinion that since the government took the burden of paying salaries for some employees in the contracted faith-based hospitals and providing subsidies for drugs and medical supplies, the FBOs were supposed to use funds which they collected from user fees and other sources prudently instead of largely depending on the government.

*“The faith-based hospitals have high expectation from the government. They want everything to be done by the government. This is not how it is supposed to be. The government is only there to compliment*” (Interview with a district health official).

Another respondent added:

*“Sometimes the district does not have funds to send to the hospital on time. If you tell them to use theirs and to be refunded, it becomes a problem. Due to this, sometimes they even go against the agreement. You may find that all of a sudden they have put a poster, for example saying that, pregnant women should come with money when they come for service. So they work impulsively, which to us is upsetting*” (Interview with a district health manager).

#### Inadequate Transparency and Mistrust Among Parties

The analysis of findings indicated that there was lack of transparency in the planning and budgeting process. Health sector public private partnership (PPP) policy guideline of 2013 and district annual planning guideline of 2011 require parties to the agreements to be involved during the preparation of district annual health plans and budgets. However, the study found that the contracted faith-based hospitals were inadequately involved. Consequently, in most cases, the contracted faith-based hospitals were not well informed of the amount of funds allocated in the district health plans to finance the contract in a particular financial year. The district officials also expressed concerns on the lack of transparency on the part of the contracted faith-based hospitals. According to the district health managers, during the hospital board meetings, the contracted hospitals did not disclose other sources of income they generated such as user fees, cost sharing, insurance and receipts in kind.

*“The hospital needs to be transparent. Our fellows (contracted hospital) do not disclose incomes generated from other sources. They only report expenditures related to basket fund. It would be good if they also disclose incomes generated from other sources*” (Interview with district health manager).

Another respondent added:

“*The hospital does not bring a full hospital revenue report. Every time for them money is not enough, but what they get is not openly put. When the basket funds delay, they immediately start complaining. Just at the beginning of the month they want money from the basket fund. If it delays, they threaten that from the following day they would start charging the patients for the services. So we usually ask them “what do you do with your money?” Why don’t you use your money and get refunded? We do not need to take their money, but even knowing their income is difficult*” (Interview with a district health manager).

#### Lack of Adequate Contract Enforcement Mechanism

At the operational level, the contracts were managed by the hospital boards which were composed of members from both the government and the contracted faith-based hospitals. The hospital board was required to hold its meeting quarterly. While in some districts meetings of the hospital boards were reported to be held, in other districts, meetings were not consistently conducted. In all the four districts of the study, SAs were supposed to be reviewed after every three to five years. However, by the time this study was conducted, no district had reviewed the SA. As a result, SAs in all study districts continued to be implemented for almost ten years without any review.

“*The contract requires that reviews be carried out annually in order to identify challenges and opportunities for further improvement. In addition, the hospital management and the district health management are required to submit progress reports to the hospital board on quarterly basis. But in my experience, these reports are rarely submitted due to either negligence or lack of understanding of the importance of these reports*” (Interview with diocese leader).

Closely related to the above, the contracted faith-based hospitals were supposed to follow the Government price list indicated in the contract when charging health services. The price list was based on the Cost Sharing Guideline of 1997. However, the review of the hospitals’ price lists as well as interviews with officials from local government and contracted FBOs revealed that the contracted hospitals were setting their own prices which were higher than the prices indicated in the contract. According to the contracted faith-based hospitals, the indicative prices were outdated and unrealistic. This was attributed to the lack of review of the SA. However, interviews with the MoH officials revealed that the Ministry was in a process of reviewing the cost sharing guide of 1997.

## Discussion

This paper aimed at understanding perceptions on contracting-out to faith-based hospitals for the provision of primary healthcare services in Tanzania. Understanding the perceptions of stakeholders is crucial for the proper implementation of SAs and could, in turn, help to assess the sustainability of SAs.

The SA was seen as an important mechanism that could be used for improving access to health services delivery, particularly in hard to reach rural areas. All faith-based hospitals included in this study were serving rural areas where public hospitals were not available. The contracted hospitals were reaching women who initially received suboptimal maternal and child healthcare services. The provision of free maternal and child health services and the subsidised user fees in the contracted faith-based hospitals, therefore, increased access to services to this hard to reach population. Previous studies have acknowledged the role of faith-based health service providers toward the UHC especially in areas where user fees are pushing majority into poverty.^23-25^

In our study, the implementation of SAs was faced by a number of challenges. In the first place, while the districts had mandate and power to get into SAs with the faith-based hospitals, they did not have financial resources to pay the contracted hospitals. They depended on the central government through basket funding which is mainly funded by the donors. The scarcity and untimely payment of funds negatively affected the implementation of the SA between the districts and the faith-based hospitals. The shortage and significant delays in the disbursement of funds from the government have led to a negative perception about contractual relationships on the side of the FBOs. While the faith-based hospitals initially appreciated the support provided by the central government, the increased number of patients coupled with the shortage and delay in the disbursement of funds from the central government increased burden to the faith-based hospitals. This situation increased the risks of disintegration of the relationship between the public and the FBOs. These findings corroborate a study of the contracting experiences in Cameroon, Chad, and Tanzania.^12^ The authors indicated that the contractual experience between the government and FBOs extended responsibilities within the context of limited financial and human resources.^12^ The findings of our study suggest that local governments need to diversify the sources of financing the implementation of the SA through alternative financing mechanisms such as community based health insurance and National Health Insurance Fund. If the funding problem is not resolved, the contractual relationship between the district and the faith-based hospitals may disintegrate in the near future.

Closely related to the above, this study revealed mistrust among the contracted parties which also affected the implementation of the contracts. It was evident that the expectations of both parties were not met. The fact that parties had higher expectations than what was achieved suggests ineffective contract designing process. This finding suggests that the contract planning process should detail the scope of the contracts and be more specific. In addition to the payment mechanisms, for instance, the contracts should clearly specify expectations of both parties and performance requirements. There is need to continually engage in dialogue involving the central and local governments, service providers and other stakeholders so as to discuss expectations and build more trust between the parties involved in SAs.

Our study found that there was low capacity in terms of contract management especially in monitoring contract implementation. It was evident from the findings that the political will to transfer authority in the contract creation and implementation to the district level was not accompanied by the capacity building of the district and local level officials. To a large extent, the districts depended on the capacity building provided by the international actors, particularly the GIZ. This support was largely provided in the two districts which were under the GIZ programme. While the institutional frameworks in terms of organisational structures were in place, they lacked adequate capacity to implement SAs. The recent assessment of the contracting experience in Tanzania reported that no need assessments were conducted prior to signing the agreement; the parties did not discharge their obligations in accordance with terms and conditions of the agreements; and that monitoring and evaluation was not adequately done by the government.^18,26^ Likewise, another study on the contractual agreement between the government and faith-based health providers in Cameroon, Tanzania, Chad, and Uganda reported limited capacity in development and implementation of contracts between the government and the FBOs.^12^

Closely connected to the above, our findings indicated inadequate contract enforcement and monitoring mechanisms. Subsequently, contracts continued to be implemented for long time without review. Lack of review resulted in hospitals setting their own prices for services offered which were often higher than those agreed in the SA. The fact that there was no review of the contracts suggests that if the contracting experience were evaluated, they would most likely fail on many levels. Studies have reported that the success of SA between the government and the NSPs often depends on their ability to create accountability.^8,17,18^ Good monitoring may ensure that the contract is followed continuously and provide information that could be used to improve services.

The findings of this study suggest that building public-sector capacity to work with the private sector, including the development of skills to negotiate and oversee contracts with private providers is imperative for achieving UHC. While the technical support provided by the development partners is highly appreciated, there are problems related to the sustainability. Over dependence on donors’ technical support also leaves the country’s domestic policy processes open to external influence resulting to a negotiated set of priorities that reflect some domestic needs and some technical, political, and economic considerations defined largely by the interests of donors. There is a strong need for the central government to build internal technical capacity in supporting the districts to establish and implement SAs with the NSPs. This will ensure that the contracting-out frameworks in place are well adapted to the local needs.

## Limitations of the Study

This study relied primarily on the review of documents and key informant interviews with various stakeholders involved in the development and implementation of SAs at the district level. First, the study did not interview service users in order to assess their experiences and perceptions on the health services provided in line with SAs. Secondly, this study did not assess the impacts of contracting-out on health systems performance such as equity, efficiency and quality. Notwithstanding these limitations, the study provides good insights on perceptions of various stakeholders on contracting-out to FBO to provide primary healthcare services in the context of poor resource settings. The lessons learned could be relevant to other countries which are implementing SAs between the government and NSPs to improve primary healthcare services.

## Conclusion

This study was one of the first assessments of the implementation of the SA between the government and the faith-based hospitals in the context of decentralisation process in Tanzania. The findings indicated that while the approach was seen as an important tool that could be used to improve access to primary healthcare services in rural and hard to reach settings, constant delays in reimbursements and lack of transparency and trust between the government and faith-based hospitals resulted into negative perceptions of the SA.

The findings of this study have some policy implications. First, the MoH should ensure timely payments to the faith-based hospitals. In addition, the local governments should diversify the sources of financing the implementation of the SA through alternative financing mechanisms such as community-based health insurance and National Health Insurance Fund. Second, there is need to continually engage in dialogue involving the central and local governments, service providers and other stakeholders so as to build more trust between partners involved in SAs. Third, the contract creation process should detail the scope of the contracts and be more specific. Last, a tailored training and continuous technical support of the central government and the umbrella organizations are needed to make sure that the contracting-out frameworks in place are well adapted to the local needs.

## Acknowledgements

This work was part of a large study on engaging NSPs towards UHC in Tanzania funded by the Alliance for Health Policy and Systems Research and Rockefeller Foundation. I am grateful to the local government officials, district health authorities, NSPs and other stakeholders in the study districts for participating in the study. I am also grateful to other research team members namely: Dr. Dereck Chitama, Prof. Esther Dungumaro and Dr. Cresencia Masawe. These team members participated in designing the study and acquisition of data.

## Ethical issues

The study was approved by the Ethical Review Committee of the National Institute of Medical Research in Tanzania (NIMR), certificate No: NIMR/HQ/R.8a/Vol.IX/1963. The study was also approved by the Ethics Review Committee (ERC) of World Health Organization. All study participants were informed of their rights and risks of participating in the study. Oral consent was obtained from all those who participated in in-depth interviews as well as all other individuals. Throughout this study, privacy and confidentiality was emphasized. All data were treated as confidential and presented only in aggregate form or anonymised. No personal identifying details of any participant were linked with the information provided by them. Access to information was limited to interviewers and the core project members.

## Competing interests

Author declares that he has no competing interests.

## Author’s contribution

SM is the single author of the paper.

## Key messages

**Implications for policy makers**

- The Ministry of Health (MoH) should ensure timely payments to the faith-based hospitals. In addition, local governments should diversify the sources of financing the implementation of the service agreement.
- The government and faith-based organizations should continually engage in dialogue so as to build more trust between the partners involved in the service agreement.
- A tailored training and continuous technical support of the central government are needed to make sure that the contracting-out frameworks in place are well adapted to the local needs.

**Implications for the public**

This study provides the public with an understanding of the process of contracting-out to faith-based hospitals to provide primary healthcare services in Tanzania, that is yet to be sufficiently evaluated. Public understanding of the process could encourage their support for the implementation of the policy.
